# Untargeted metabolomics for acute intra-abdominal infection diagnosis in serum and urine using UHPLC-TripleTOF MS

**DOI:** 10.3389/fmolb.2025.1534102

**Published:** 2025-05-08

**Authors:** Zhenhua Dong, Shaopeng Zhang, Hongwei Zhang, Dingliang Zhao, Ziwen Pan, Daguang Wang

**Affiliations:** ^1^ Gastric and Colorectal Surgery Department, The First Hospital of Jilin University, Changchun, Jilin, China; ^2^ Second Urology Department, The First Hospital of Jilin University, Changchun, Jilin, China

**Keywords:** UHPLC-TripleTOF MS, acute intra-abdominal infection, metabolomics, biomarkers, serum, urine

## Abstract

**Introduction:**

Acute intra-abdominal infection (IAI) is a prevalent and life-threatening condition in general surgery, with significant implications for patient mortality. However, the timely identification of IAI is often hindered by the limitations of current medical laboratory sciences and imaging diagnostics.

**Methods:**

To address this critical issue, we employed metabolomics to identify early biomarkers for IAI. In this study, we enrolled a cohort of 30 IAI patients and 20 healthy volunteers. Following preliminary experimental processing, all serum and urinary samples were subjected to ultrahigh performance liquid chromatography-triple time-of-flight mass spectrometry analysis. Initial metabolite profiling was conducted using total ion current chromatography and principal component analysis. Differential metabolites were subsequently identified through Student's t-test, partial least squares discriminant analysis, and support vector machine. Hierarchical clustering analysis was then applied to assess the discriminatory power of the selected metabolites. Based on receiver operating characteristic curve analysis, we identified the most promising biomarkers, which were further subjected to enrichment analysis. Additionally, we stratified patients according to the severity and etiology of IAI to explore potential differences among these subgroups.

**Results:**

Our findings revealed five serum and two urinary metabolites as potential biomarkers for IAI. The serum biomarkers were associated with the Fatty Acid Biosynthesis pathway, while the urinary biomarkers were linked to the Catecholamine Biosynthesis pathway. Notably, no significant differences were observed among the three types of IAI or the seven etiologies studied.

**Discussion:**

For individuals at risk of IAI, regular screening of these biomarkers could facilitate the early and convenient identification of the condition, thereby improving patient outcomes.

## 1 Introduction

Acute intra-abdominal infection (IAI), a prevalent and clinically significant condition in general surgery, is a major contributor to patient mortality (Huston et al., 2024). IAI primarily results from bacterial invasion of the peritoneum, retroperitoneum, or intra-abdominal organs (Sartelli et al., 2024). It is frequently secondary to various underlying conditions, including acute appendicitis (AA) (Di Saverio et al., 2020), acute gastrointestinal perforation (AGP), acute cholecystitis, and other related pathologies. The hallmark clinical manifestations of IAI typically include acute-onset abdominal pain accompanied by localized or systemic inflammatory responses (Sartelli et al., 2021). If not promptly and effectively managed, IAI can trigger a cascade of inflammatory responses, potentially escalating into a cytokine storm and subsequent sepsis. Globally, there are approximately 50 million sepsis cases and 11 million deaths related to sepsis annually, representing a significant public health challenge and imposing a substantial socioeconomic burden on healthcare systems worldwide (Rudd et al., 2020). Effective management of IAI hinges on early recognition, timely source control, and the judicious use of antibiotics combined with fluid resuscitation in severe cases, highlighting the critical role of early diagnosis in improving patient outcomes (Wu et al., 2020).

Although C-reactive protein (CRP) and procalcitonin (PCT) are commonly utilized biomarkers for infection assessment in clinical practice, their diagnostic performance for AA remains suboptimal, with sensitivity and specificity ranging from 67% to 97.8% and 31.9%–80%, respectively (Moris et al., 2021). Furthermore, PCT has been reported to exhibit limited sensitivity in detecting localized inflammation (Martínez-Sagasti et al., 2018). Ultrasonography is recommended as the primary imaging modality for detecting IAI owing to its high sensitivity and specificity (Perrone et al., 2020). However, its diagnostic accuracy can be compromised by various factors, including the presence of free gas, patient obesity, and the operator’s technical expertise. Computed tomography provides superior diagnostic performance with more comprehensive imaging capabilities (Wang K. et al., 2021); however, its widespread application is constrained by increased radiation exposure and higher costs (Smoll et al., 2023). Therefore, the identification and validation of specific biomarkers for the early detection of IAI hold critical clinical significance.

Untargeted metabolomics, a rapidly evolving field in the post-genomic era, offers significant potential for elucidating complex disease mechanisms through the comprehensive analysis of low-molecular-weight compounds in biological samples (Qiu et al., 2023; Saoi and Britz-McKibbin, 2021). We selected metabolomics as the core methodology for this study due to its demonstrated diagnostic utility and essential role in identifying biomarkers for various diseases, including gastric cancer, colorectal cancer (Gumpenberger et al., 2021; Zhang et al., 2022), and breast cancer (Long et al., 2021; Subramani et al., 2022). Here, an ultrahigh performance liquid chromatography-triple time-of-flight mass spectrometry (UHPLC-TripleTOF MS) method is reported to elucidate potential biomarkers from serum and urine to enable the early detection of IAI, ultimately striving to enhance patient outcomes and improve prognosis.

## 2 Methods

### 2.1 Patients

The study protocol was approved by the Institutional Review Board of The First Hospital of Jilin University (Approval No. 24K181-001), and written informed consent was obtained from all participating patients prior to their enrollment in the study. The study was conducted in full compliance with international ethical standards and guidelines for biomedical research involving human subjects. Serum and urine specimens were prospectively collected from consecutive patients with pathologically confirmed IAI who were admitted to the Department of Gastrointestinal Surgery at The First Hospital of Jilin University during the study period from July 2024 to September 2024. The study cohort comprised 30 consecutively enrolled patients with confirmed intra-abdominal infections and 20 age- and sex-matched healthy controls. The inclusion criteria for patients were (1) postoperative pathology-confirmed AA or AGP; (2) no history of tumors, hormone treatment, radiotherapy, or chemotherapy; (3) preserved bone marrow, liver, renal, and heart functions; and (4) absence of overt acute lung inflammation. The inclusion criteria for healthy controls were as follows: individuals with normal results in blood routine, urine routine, liver function, renal function, and electrocardiogram tests. The exclusion criteria for healthy controls were defined as follows: (1) congenital diseases; (2) metabolic diseases (e.g., diabetes); or mental illnesses; (3) pregnant or lactating women; (4) individuals with recent intoxication, drug abuse, or use of proton pump inhibitors, hormones, or nonsteroidal anti-inflammatory drugs; (5) acute or chronic inflammation; (6) major stress responses (e.g., burns or psychic trauma) within the past 2 weeks; or (7) blood disorders including leukemia or anemia. The exclusion criteria for IAI resemble the ones for healthy people, except for acute inflammation.

For subjects, we collected their basic information including age and sex; basic medical histories including surgical history and hypertension; routine blood examination including the number of white blood cells (WBC), neutrophilicgranulocytes (NE), lymphocytes, monocytes, red blood cells (RBC), platelets, and hemoglobin; liver function test including total bilirubin, alanine transaminase, aspartate aminotransferase, total bile acid, and albumin; renal function tests including blood urea nitrogen and serum creatinine; and inflammatory indexes including the percentage of NE, CRP, and PCT. The data for continuous variables are presented as mean ± standard deviation. Statistical comparisons between the healthy and IAI groups were performed using the following approach: First, Levene’s test was conducted to assess the homogeneity of variance. Based on the Levene’s test results, either Student’s t-test (for equal variances) or Welch’s corrected t-test (for unequal variances) was subsequently applied to evaluate the intergroup differences. Data for categorical variables are expressed as numbers (percentages). Statistical analysis was performed according to the following criteria: (1) the Pearson chi-square test was applied when the sample size (n) was ≥40 and all theoretical frequencies were >5; (2) Yates’ corrected chi-square test was used when n was ≥40 and at least one theoretical frequency was between 1 and 5; and (3) Fisher’s exact test was employed when n was <40 or any theoretical frequency was <1.

### 2.2 Sample collection

Biological specimens were collected under standardized conditions: serum and urine samples from patients were obtained precisely 1 hour before emergency surgical intervention, while corresponding samples from healthy controls were collected following an overnight fast during morning hours (8:00–10:00 a.m.). Following collection, all biological specimens were immediately processed through centrifugation at 3,500 g for 10 min at 4°C. The resulting supernatant was aliquoted into sterile cryovials and rapidly transferred to −80°C ultra-low temperature freezers for long-term storage until subsequent biochemical analyses. For quality control purposes, an aliquot of 30 μL from each plasma sample was systematically pooled to create a representative quality control (QC) sample, which was subsequently processed in parallel with experimental samples throughout the analytical procedures. Subsequently, 100-μL aliquots of plasma were precisely transferred into 1.5-mL microcentrifuge tubes and thoroughly mixed with 300 μL of ice-cold methanol:acetonitrile solution (1:1, v/v). The resulting mixture was vigorously vortexed for 1 min at 1,000 × g and subsequently incubated at 4°C for 20 min to facilitate complete protein precipitation and metabolite extraction. Afterward, the samples were centrifuged at 13,500 × g for 10 min at 4°C. The resulting supernatant was carefully collected and filtered through a 0.22-μm PVDF membrane using a syringe filter, and a 100-μL aliquot was transferred to an LC-MS vial for subsequent mass spectrometry analysis. For urine sample collection, clean-catch midstream specimens were obtained following standard clinical protocols. The subsequent processing steps, including centrifugation, aliquoting, and storage conditions, were maintained consistent with the serum sample protocol to ensure methodological uniformity across sample types.

### 2.3 UHPLC-TripleTOF MS

A Sciex TripleTOF MS 5600+ (Sciex, Framingham, MA, United States) and a Shimadzu Exion UHPLC (Shimadzu, Kyoto, Japan) were used for the UHPLC-TripleTOF MS analysis. We used a Vortex 3 mixer from Germany (IKA) and an H165R centrifuge at 4°C from Xiangyi Centrifuge Instrument Co., Ltd. (China) for sample processing. Solvents included acetonitrile (HPLC-grade, Sigma-Aldrich, United States), formic acid (HPLC-grade, Sigma-Aldrich, United States), methanol (HPLC-grade, Merck, Germany), and deionized water (Watson, China). APCI Positive and Negative Calibration Solutions (Thermo Fisher Scientific, Waltham, Massachusetts, United States) were used for ion calibration. The Exion UHPLC System served as the liquid chromatography system, with an Acquity UPLC HSS T3 column (2.1 mm × 100 mm, 1.8 μm; Waters Corporation, Milford, MA, United States) set at 35°C. The mobile phase comprised solvent A (0.1% formic acid in water) and solvent B (acetonitrile). A 5-min equilibration with the initial mobile phase was performed to ensure system stability. To ensure system stability and method reproducibility, an initial set of ten quality control (QC) samples was analyzed prior to experimental sample runs. Following the completion of each batch run, principal component analysis (PCA) analysis was conducted to evaluate the data quality. The batch was accepted only when the QC samples demonstrated tight clustering in the PCA plot; otherwise, the data were rejected. The mobile phase was delivered at a constant flow rate of 0.35 mL/min throughout the chromatographic separation, with a precise injection volume of 0.5 μL maintained for all analytical runs. The chromatographic separation was achieved using the following gradient elution program: 0.5 min (A phase 98% + B phase 2%); 1.5 min (A phase 80% + B phase 20%); 4 min (A phase 35% + B phase 65%); 11 min (A phase 5% + B phase 95%); 15 min (A phase 5% + B phase 95%); 15.1 min (A phase 98% + B phase 2%); and 20 min (A phase 98% + B phase 2%). The total run time was 20 min.

The UHPLC-TripleTOF MS was equipped with an electrospray ionization source operated in positive and negative ionization modes, allowing high-resolution mass spectrometry metabolite detection and characterization. The first-level mass spectrometry (MS1) parameters were optimized as follows: for positive ion mode, ion spray voltage 5500 V, temperature 550°C, gas 1 (nitrogen) and gas 2 (nitrogen) at 55 psi, curtain gas (nitrogen) at 30 psi, declustering potential 100 V, and collision energy 10 eV; for negative ion mode, ion spray voltage −5500 V, declustering potential −100 V, and collision energy −10 eV, while maintaining all other parameters consistent with the positive ion mode configuration. The second-level mass spectrometry (MS2) parameters were configured as follows: for positive ion mode, ion spray voltage 5500 V, temperature 550°C, gas 1 and gas 2 at 55 psi, curtain gas at 30 psi, declustering potential 100 V, collision energy 35 eV, collision energy spread 15 eV, ion release delay 67 V, and ion release width 25 V; for negative ion mode, ion spray voltage −4500 V, declustering potential −100 V, and collision energy −35 eV, while keeping the remaining parameters in accordance with the positive ion mode configuration. MS1, with a mass range of m/z 50–1,500, focuses on detecting and quantifying intact ions, providing a broad metabolic profile. MS2, with a mass range of m/z 50–1,000, delves deeper into the structural details of selected ions, enabling precise identification and validation of molecular structures. Together, MS1 and MS2 form the basis of tandem mass spectrometry, which is essential for untargeted metabolomics and proteomics, as it combines broad detection with detailed structural analysis.

### 2.4 Statistical analysis

The raw high-resolution mass spectrometry data were processed and analyzed using dedicated software packages: Analyst TF 1.7.1 (United States) was employed for data acquisition and conversion to *.wiff format, while PeakView 2.2 software (United States) was utilized for total ion current (TIC) generation and preliminary data evaluation. Following comprehensive data preprocessing, including standardization, normalization, noise filtration, and peak alignment procedures, PCA was performed using MetaboAnalyst 6.0 (https://www.metaboanalyst.ca), a web-based platform for metabolomics data analysis that could identify potential metabolic patterns and sample clustering. Subsequently, univariate and multivariate statistical analyses were conducted using MetaboAnalyst 6.0, including Student’s t-test for individual metabolite comparisons and partial least squares discriminant analysis (PLS-DA) with variable importance in projection (VIP) scoring to identify significantly differentiated metabolites between experimental groups. Following false discovery rate (FDR) correction for multiple T-tests using the Benjamini–Hochberg method, statistically significant metabolites were selected based on dual criteria: adjusted p-value threshold of <0.05 and VIP score exceeding 1.0. Furthermore, BRB-ArrayTools (version 4.6.1) was utilized to conduct support vector machine (SVM) analysis, enabling the selection of discriminatory metabolites based on their classification weight functions, with optimal model performance achieving 100% classification accuracy in sample differentiation. Subsequently, metabolite identification was performed by querying the Human Metabolome Database (HMDB, version 5.0) using precise mass-to-charge (m/z) ratios, with structural annotation and nomenclature assignment conducted for matched entries while excluding unidentified or ambiguously characterized metabolites lacking sufficient spectral or biochemical information. Following metabolite identification, hierarchical cluster analysis (HCA) was performed using RStudio (version 2023.09.1, build 494) and R (version 4.3.1, 2023.06.16, ucrt) (Boston, United States, https://www.rstudio.com) with the “ggplot2” and “pheatmap” package for visualization, while receiver operating characteristics (ROC) curve analysis was conducted using SPSS Statistics (version 27.0) to evaluate the diagnostic potential of identified metabolites.

Metabolites demonstrating superior diagnostic performance, as evidenced by an area under the curve (AUC) exceeding 0.9 or less than 0.1 in ROC analysis, were identified as potential biomarkers for further validation. Subsequently, comparative boxplot visualization was performed using R Studio with the ggplot2 package (version 3.4.2) to statistically represent the distribution characteristics of metabolites, including median values, interquartile ranges (IQR), and outlier detection, between the healthy control group and the IAI patient group.

### 2.5 Enrichment analysis

Following the selection of metabolites by SVM in serum and urine samples, we retrieved the HMDB numbers of each metabolite associated with inflammation. These HMDB numbers were then input into the enrichment module of MetaboAnalyst 6.0. Subsequently, an enrichment plot was generated to visualize the enrichment results. Pathways with a p-value >0.05 were considered to have potential statistical significance, whereas those with a p-value <0.05 were deemed statistically significant.

### 2.6 Comparison of diagnostic performance of inflammatory markers

For IAI, common inflammatory markers include CRP, WBC, PCT, and NE. We compared the diagnostic efficacy between these conventional indicators and the identified biomarkers using ROC analysis. Additionally, we included statistically significant indexes from the baseline characteristics table in the ROC analysis. If the identified metabolites exhibited a larger AUC, they demonstrated higher accuracy in detecting IAI.'

### 2.7 Subgroup analysis

Given the heterogeneity of the IAI etiology, we stratify patients based on the severity of IAI. IAI guidelines classify the condition into three grades: mild infection, medium infection, and severe infection. A mild infection is localized with no systemic toxic symptoms, and vital signs are stable. A medium infection has spread, accompanied by systemic toxic symptoms (such as fever or increased heart rate), but without organ dysfunction. A severe infection is widespread and accompanied by significant systemic toxic symptoms and organ dysfunction (such as hypotension or respiratory failure). All IAI cases should be categorized into one of the three grades, and PCA was performed to examine differences in metabolic patterns and sample clustering among the three groups. Subsequently, based on postoperative pathology findings, all IAI cases were further classified into different etiological subtypes. PCA was also conducted to investigate variations in metabolic patterns and sample clustering across these etiological categories. If these groups were not distinctly separated in the PCA plot, this may suggest that the metabolic changes associated with inflammation exhibit similarities among different grades and etiologies.

## 3 Results

### 3.1 Subject characteristics

This study enrolled 20 healthy controls, designated as NG1 to NG20, and 30 IAI cases, labeled as AP1 to AP30. Based on postoperative pathology results, the IAI cases comprised five cases of gastric ulcers, three cases of duodenal ulcers, three cases of small intestinal trauma, four cases of colon diverticular perforations, five cases of acute non-suppurative appendicitis, six cases of acute suppurative appendicitis, and four cases of gangrenous perforated appendicitis. The age of the 20 healthy volunteers was 55.85 ± 9.07 years; the age of the 30 IAI patients was 56.90 ± 7.99 years. The IAI group included ten women and twenty men, whereas the healthy control group consisted of ten women and ten men. Compared with healthy controls, we observed statistically significant changes in seven indexes. Among these, WBC (p < 0.001), NE (p < 0.001), PCT (p < 0.001), CRP (p < 0.001), and bile acids (p < 0.001) were significantly increased, while lymphocyte (p < 0.001) and albumin (p = 0.001) were significantly decreased (Table 1). These indexes will be incorporated into the analysis to compare their diagnostic performance with the identified biomarkers.

**TABLE 1:** The basic information about 50 participants.

	Healthy people (20)	IAI patients (30)	p-value
Sex, n (%)			0.239
Male	10 (50%)	20 (66.7%)	
Hypertension, n (%)	2 (10%)	4 (13.3%)	0.999
Surgical history, n (%)	2 (10%)	5 (16.7%)	0.803
Age	55.9 ± 9.07	56.9 ± 7.99	0.785
WBC (10^9^/L)	5.90 ± 1.14	12.3 ± 5.19	<0.001
MO (10^9^/L)	0.400 ± 0.120	0.680 ± 0.410	0.060
NE (10^9^/L)	3.36 ± 0.860	9.05 ± 3.70	<0.001
LY (10^9^/L)	1.99 ± 0.520	1.01 ± 0.590	<0.001
PLT (10^9^/L)	229 ± 73.9	251 ± 108	0.426
RBC (10^12^/L)	4.56 ± 0.570	4.43 ± 0.580	0.424
HGB (g/L)	140 ± 18.9	133 ± 22.2	0.257
CRP (mg/L)	0.590 ± 0.270	94.7 ± 75.6	<0.001
PCT (ng/ml)	0.320 ± 0.130	5.60 ± 6.36	<0.001
AST (U/L)	23.0 ± 8.54	20.3 ± 11.7	0.392
ALT (U/L)	19.8 ± 15.4	17.1 ± 15.5	0.544
Creatinine (umol/L)	91.2 ± 108	75.3 ± 33.5	0.454
Urea nitrogen (mmol/L)	5.43 ± 1.47	7.05 ± 3.45	0.055
Total bilirubin (umol/L)	13.8 ± 5.98	16.1 ± 7.68	0.257
Bile acids (umol/L)	3.80 ± 1.66	15.5 ± 17.5	0.001
Albumin (g/L)	43.9 ± 3.15	31.5 ± 11.1	0.001

Abbreviation:

Alanine transaminase (AST), aspartate aminotransferase (ALT), C-reactive protein (CRP), hemoglobin (HGB), lymphocyte (LY), monocyte (MO), neutrophilicgranulocyte (NE), platelet (PLT), procalcitonin (PCT), red blood cells (RBC), and white blood cells (WBCs).

### 3.2 Serum sample

**FIGURE 1:**
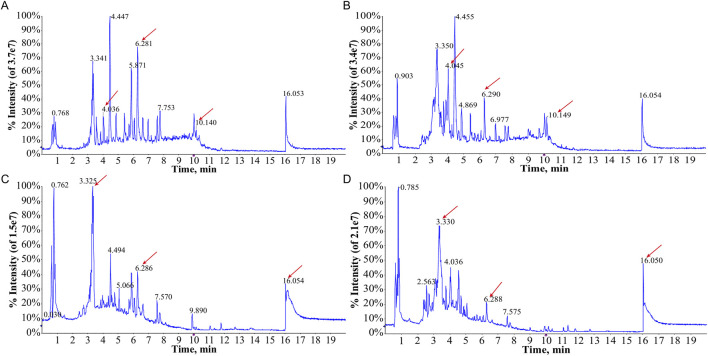
Total ion current (TIC) chromatograms of metabolites of serum samples from the healthy group and the IAI group. **(A)** TIC profiles of the healthy group in the positive ion mode; **(B)** TIC profiles of the IAI group in the positive ion mode; **(C)** TIC profiles of the healthy group in the negative ion mode; **(D)** TIC profiles of the IAI group in the negative ion mode. Red arrows indicate metabolites subsequently identified as having different abundances in the two groups. Abbreviations: IAI, Acute intra-abdominal infection.

**FIGURE 2:**
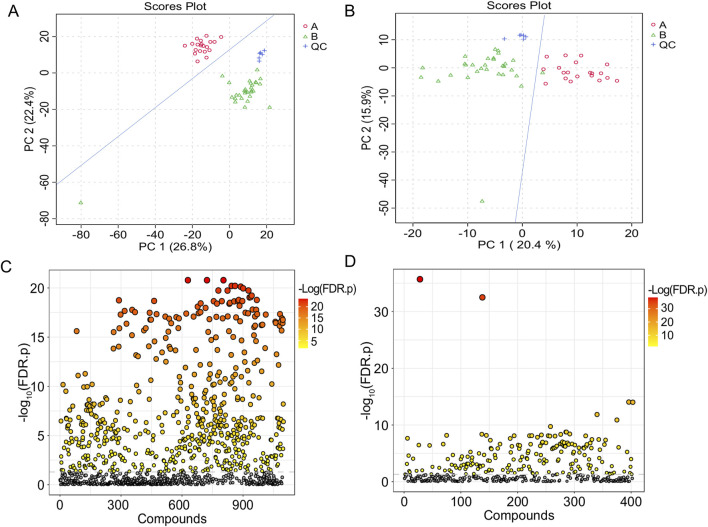
T-test and PCA of serum samples from the healthy group and the IAI group. **(A)** PCA in the positive ion mode; **(B)** PCA in the negative ion mode; each dot represents a single sample; **(C)** t-test in the positive ion mode; **(D)** t-test in the negative ion mode. The vertical coordinate is the p-value, the horizontal coordinate is the level of each metabolite, and the orange represents the significant metabolites, with p< 0.05. Abbreviations: IAI, Acute intra-abdominal infection; A, healthy group; B, IAI group; QC, quality control group; PCA, principal component analysis.

The TIC of serum samples from IAI patients and healthy volunteers were analyzed in both positive and negative ionization modes. The TIC profiles demonstrated notable variations in peak intensities for multiple metabolites between the two groups, suggesting the presence of distinct differential metabolites (Figure 1). PCA was performed on serum samples from IAI patients, healthy volunteers, and quality controls (QC) in both positive and negative modes (Figure 2). In the PCA analysis, each point on the plot represents an individual sample, where greater distances between points reflect larger differences in metabolite profiles, while closer distances indicate smaller differences. A close clustering of samples within each group in the PCA plot suggests low intra-group variability, whereas the separation between the three groups highlights significant intergroup differences. Additionally, the tight clustering of QC samples further validates the consistency of the experimental data.

**FIGURE 3:**
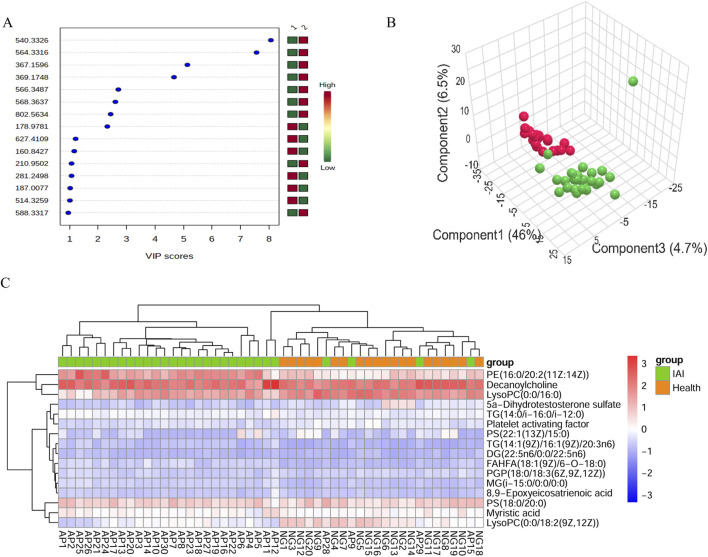
VIP score plot, PLS-DA plot, and clustering analysis of serum samples from the healthy group and the IAI group in positive and negative models. **(A)** VIP score plot for the selected differential metabolites with VIP >1, 1, healthy group; 2, IAI group.; **(B)** PLS-DA plot, red balls, healthy group; green balls, IAI group; **(C)** hierarchical clustering analysis of the 16 differential metabolites. The color scale represents the relative abundance of metabolites, with red indicating higher abundance, white indicating zero abundance, and blue indicating lower abundance. The right side of the figure shows the peak name of each metabolite, while the dendrogram on the left and top represents the clustering results of the differential metabolites. The sample numbers are shown at the bottom of the figure. Abbreviations: VIP, variable importance in projection; PLS-DA, partial least squares discriminant analysis; IAI, Acute intra-abdominal infection.

Following FDR correction, a comparative analysis between IAI patients and healthy volunteers using Student’s t-test (p < 0.05) revealed significant differences in 431 metabolites in the positive ionization mode and 216 metabolites in the negative ionization mode (Figure 2 and Supplementary Sheet S1). To further refine the selection, PLS-DA was applied, resulting in the identification of 51 metabolites with VIP scores >1 in both positive and negative ionization modes (Figure 3 and Supplementary Sheet S2). The PLS-DA plot demonstrates a clear separation between the two groups (Figure 3), highlighting the potential of these metabolites to serve as biomarkers for IAI.

SVM analysis was conducted for further refinement, identifying 20 metabolites with 100% weight in the models: 11 in the positive ionization mode and nine in the negative ionization mode. Based on the HMDB, http://www.hmdb.ca, we identified 16 metabolites that met our predefined criteria: myristic acid, PGP(18:0/18:3 (6Z,9Z,12Z)), TG(14:1 (9Z)/16:1 (9Z)/20:3n6), decanoylcholine, MG(i-15:0/0:0/0:0), TG(14:0/i-16:0/i-12:0), DG(22:5n6/0:0/22:5n6), PS(18:0/20:0), PE(16:0/20:2 (11Z:14Z)), FAHFA(18:1 (9Z)/6-O-18:0), 8,9 epoxyeicosatrienoic acid, 5a-dihydrotestosterone sulfate, lysoPC(0:0/16:0), lysoPC(0:0/18:2 (9Z,12Z)), platelet-activating factor, and PS(22:1 (13Z)/15:0) (Table 2). Using the 16 identified metabolites, we employed Support Vector Machine Discriminant Analysis (SVM-DA) to classify 28 of 30 IAI cases as IAI and 20 of 20 healthy individuals as healthy, as expected, achieving a sensitivity of 93.3% and a specificity of 100%.

**TABLE 2:** Sixteen differential metabolites in serum samples between the healthy and IAI groups.

Ionization	Mass-to-charge ratio (*m/z*)	Chemical formula	Metabolite
ESI+	246.2441	C14H28O2	Myristic acid
ESI+	285.1718	C44H72O15P2	PGP(18:0/18:3(6Z,9Z,12Z))
ESI+	301.2031	C53H92O6	TG(14:1(9Z)/16:1(9Z)/20:3n6)
ESI+	318.3017	C15H32NO2+	Decanoylcholine
ESI+	334.2961	C18H36O4	MG(i-15:0/0:0/0:0)
ESI+	362.3278	C49H94O6	TG(14:0/i-16:0/i-12:0)
ESI+	758.5726	C47H72O5	DG(22:5n6/0:0/22:5n6)
ESI+	784.587	C44H80NO10P	PS(18:0/20:0)
ESI+	806.5728	C41H78NO8P	PE(16:0/20:2(11Z:14Z)
ESI-	281.2498	C36H68O4	FAHFA(18:1(9Z)/6-O-18:0)
ESI-	339.2343	C20H32O3	8,9-Epoxyeicosatrienoic acid
ESI-	369.1748	C19H30O5S	5a-Dihydrotestosterone sulfate
ESI-	540.3326	C24H50NO7P	LysoPC(0:0/16:0)
ESI-	564.3316	C26H50NO7P	LysoPC(0:0/18:2(9Z,12Z))
ESI-	568.3637	C26H54NO7P	Platelet-activating factor
ESI-	802.5634	C43H82NO10P	PS(22:1(13Z)/15:0)

Hierarchical clustering analysis was performed on the 16 metabolites to evaluate the similarity of differential metabolites (represented by the dendritic structure on the left side of the figure) and samples (represented by the dendritic structure above the figure) (Figure 3). The color intensity in the heatmap reflects the relative abundance of metabolites in the samples, with red indicating the highest content and blue representing the lowest content. The figure demonstrates that the 16 metabolites possess sufficient discriminative power to clearly separate the 50 samples into IAI and healthy groups.

**FIGURE 4:**
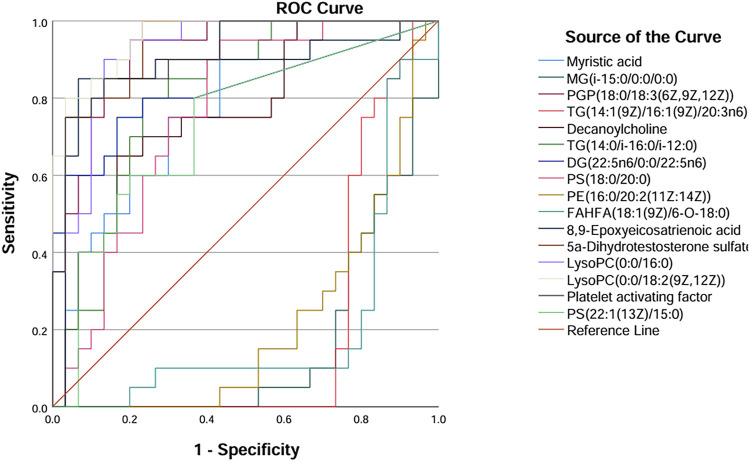
ROC curves of sixteen metabolites in serum samples. Abbreviations: ROC, receiver operating characteristics.

**FIGURE 5:**
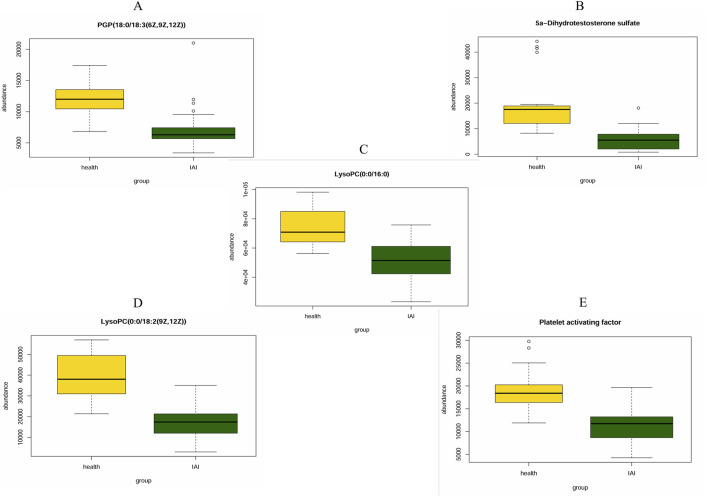
Mean plots of five differential serum metabolites between the healthy group and the IAI group. **(A)** PGP (18:0/18:3 (6Z,9Z,12Z)), **(B)** 5a-Dihydrotestosterone sulfate, **(C)** LysoPC(0:0/16:0), **(D)** LysoPC (0:0/18:2 (9Z,12Z)), and **(E)** platelet activating factor. Abbreviations: IAI, acute intra-abdominal infection.

ROC curves were generated for the 16 metabolites, and five metabolites with AUC> 0.9 were selected for further analysis, namely PGP(18:0/18:3 (6Z,9Z,12Z)), 5a-dihydrotestosterone sulfate, lysoPC(0:0/16:0), lysoPC(0:0/18:2 (9Z,12Z)), and platelet-activating factor (Figure 4; Table 3). Boxplots of the five biomarkers were constructed to visualize the trends in metabolite levels between the two groups. Compared with healthy controls, the mean levels of the five biomarkers were significantly reduced in the IAI group (Figure 5).

**TABLE 3:** The AUC value of 16 metabolites between two groups.

Variable	AUC	SE	95% confidence intervals	
			lower	upper
Myristic acid	0.797	0.062	0.675	0.918
MG(i-15:0/0:0/0:0)	0.162	0.055	0.053	0.270
PGP(18:0/18:3(6Z,9Z,12Z))	0.908	0.043	0.824	0.993
TG(14:1(9Z)/16:1(9Z)/20:3n6)	0.202	0.068	0.069	0.334
Decanoylcholine	0.762	0.069	0.627	0.897
TG(14:0/i-16:0/i-12:0)	0.805	0.062	0.684	0.926
DG(22:5n6/0:0/22:5n6)	0.823	0.067	0.693	0.954
PS(18:0/20:0)	0.758	0.068	0.625	0.891
PE(16:0/20:2(11Z:14Z))	0.215	0.064	0.090	0.340
FAHFA(18:1(9Z)/6-O-18:0)	0.202	0.068	0.067	0.336
8,9-Epoxyeicosatrienoic acid	0.873	0.060	0.755	0.992
5a-Dihydrotestosterone sulfate	0.937	0.032	0.873	1.000
LysoPC(0:0/16:0)	0.923	0.037	0.851	0.996
LysoPC(0:0/18:2(9Z,12Z))	0.960	0.023	0.915	1.005
Platelet-activating factor	0.917	0.040	0.839	0.994

Area under the curve, AUC

### 3.3 Urine sample

**FIGURE 6:**
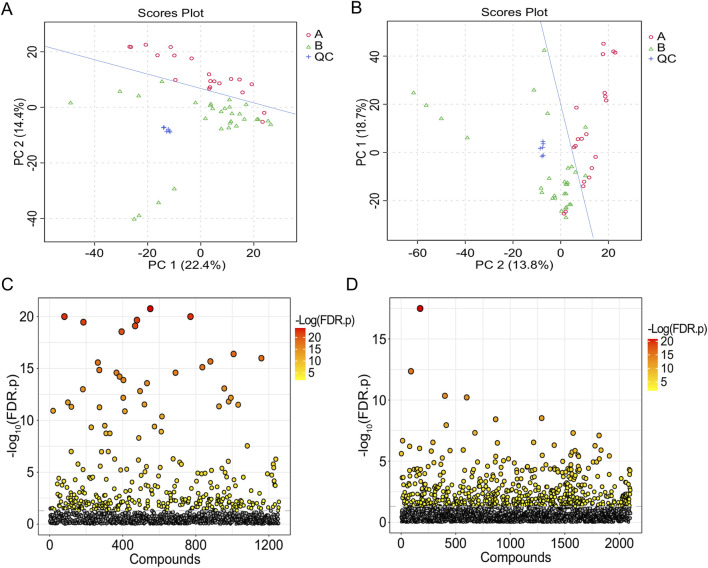
T-test and PCA of urine samples from the healthy group and the IAI group. **(A)** PCA in the positive ion mode, **(B)** PCA in the negative ion mode (each dot represents a single sample), **(C)** t-test in the positive ion mode, and **(D)** t-test in the negative ion mode. The vertical coordinate is the p-value, the horizontal coordinate is the level of each metabolite, and the orange represents the significant metabolites, with p< 0.05. Abbreviations: IAI, acute intra-abdominal infection; A, healthy group; B, IAI group; QC, quality control group; PCA, principal component analysis.

**FIGURE 7:**
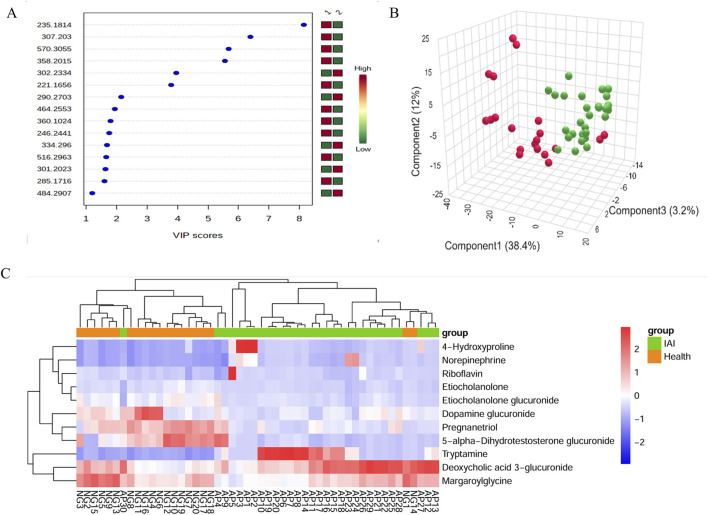
VIP score plot, PLS-DA plot, and clustering analysis of urine samples from the healthy group and the IAI group in positive and negative models. **(A)** VIP score plot for the selected differential metabolites with VIP >1, 1, healthy group; 2, IAI group; **(B)** PLS-DA plot, red balls, healthy group; green balls, IAI group; **(C)** hierarchical clustering analysis of the 16 differential metabolites. The color scale represents the relative abundance of metabolites, with red indicating higher abundance, white indicating zero abundance, and blue indicating lower abundance. The right side of the figure shows the peak name of each metabolite, while the dendrogram on the left and top represents the clustering results of the differential metabolites. The sample numbers are shown at the bottom of the figure. Abbreviations: VIP, variable importance in projection; PLS-DA, partial least squares discriminant analysis; IAI, acute intra-abdominal infection.

The analytical procedure for urine samples was consistent with that of serum samples. The TIC revealed differences in the intensity of certain metabolites in urine samples at the same retention time between the healthy and IAI groups in both positive and negative ionization modes (Supplementary Figure S1). In the PCA, we observed minimal intra-group variability, while significant intergroup differences were evident. The consistent clustering of QC samples confirms the reliability of the experimental data (Figure 6). Following the FDR correction for the T-test, we identified 269 metabolites with significant differences in the positive ionization mode and 409 metabolites in the negative ionization mode (Figure 6, Supplementary Sheet S3). We employed PLS-DA for further screening, selecting 63 metabolites with VIP scores >1 in both positive and negative ionization modes (Figure 7, Supplementary Sheet S4). In the PLS-DA plot, the two groups could be broadly separated, indicating distinct metabolic profiles of approximately 63 metabolites (Figure 7).

SVM was applied to identify 12 metabolites with a weight of 100%. Subsequently, the m/z of the metabolites were queried against the HMDB (http://www.hmdb.ca); 11 metabolites matched the predefined criteria based on accurate mass and charge ratio: tryptamine, etiocholanolone, deoxycholic acid 3-glucuronide, margaroylglycine, norepinephrine, etiocholanolone glucuronide, 4-hydroxyproline, dopamine glucuronide, riboflavin, pregnanetriol, and 5-alpha-dihydrotestosterone glucuronide (Table 4). We employed SVM-DA on the 11 metabolites to classify the samples, and the results indicate, as expected, a specificity of 85% and a sensitivity of 86.7%.

**TABLE 4:** Eleven metabolites differentially expressed in the urine samples of the healthy and IAI groups.

Ionization	Mass-to-charge ratio (*m/z*)	Chemical formula	Metabolite
ESI+	221.1656	C10H12N2	Tryptamine
ESI+	273.2219	C19H30O2	Etiocholanolone
ESI+	285.1716	C30H48O10	Deoxycholic acid 3-glucuronide
ESI+	334.296	C19H37NO3	Margaroylglycine
ESI+	152.0709	C8H11NO3	Norepinephrine
ESI+	484.2907	C25H38O8	Etiocholanolone glucuronide
ESI-	150.0567	C5H9NO3	4-Hydroxyproline
ESI-	350.0884	C14H19NO8	Dopamine glucuronide
ESI-	375.1307	C17H50N4O6	Riboflavin
ESI-	931.5049	C25H38O8	5-alpha-Dihydrotestosterone glucuronide

**FIGURE 8:**
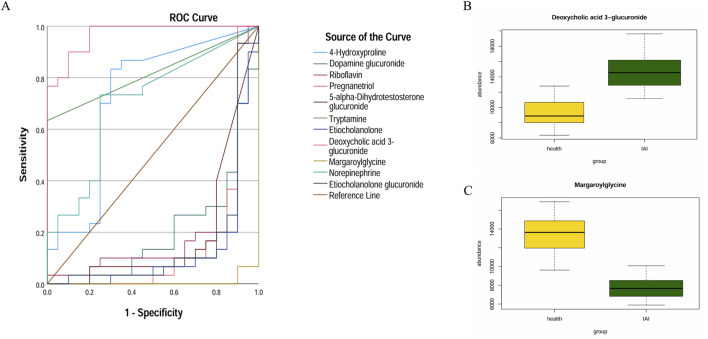
ROC curves and mean plots of urine metabolites between the healthy group and the IAI group. **(A)** ROC curves of the 11 metabolites, **(B)** mean plot of deoxycholic acid 3-glucuronide, and **(C)** mean plot of metabolite margaroylglycine. Abbreviations: ROC, receiver operating characteristics; IAI, Acute intra-abdominal infection.

Hierarchical clustering analysis revealed that the 11 metabolites exhibit excellent discriminative capability, effectively distinguishing between two groups (Figure 7). Finally, we protract ROC for 11 metabolites and selected deoxycholic acid 3-glucuronide and margaroylglycine as potential urine biomarkers, with AUC> 0.9 and AUC< 0.1, respectively (Figure 8; Table 5). We constructed boxplots of the two biomarkers to visualize the trends in metabolite levels between the two groups. Compared with healthy controls, the mean level of the deoxycholic acid 3-glucuronide was significantly increased, and margaroylglycine was significantly decreased in the IAI group (Figure 8).

**TABLE 5:** The AUC value of 11 metabolites between two groups.

Variable	AUC	SE	95% confidence intervals	
			lower	upper
4-Hydroxyproline	0.725	0.081	0.567	0.883
Riboflavin	0.195	0.073	0.052	0.338
Pregnanetriol	0.155	0.069	0.019	0.291
5-alpha-Dihydrotestosterone glucuronide	0.19	0.069	0.054	0.326
Dopamine glucuronide	0.2	0.065	0.073	0.327
Tryptamine	0.817	0.06	0.699	0.934
Etiocholanolone	0.127	0.059	0.012	0.241
Deoxycholic acid 3-glucuronide	0.968	0.02	0.929	1.008
Margaroylglycine	0.007	0.007	-0.007	0.021
Norepinephrine	0.706	0.076	0.557	0.855
Etiocholanolone glucuronide	0.155	0.067	0.023	0.287

Area under the curve, AUC

### 3.4 Enrichment analysis

**FIGURE 9:**
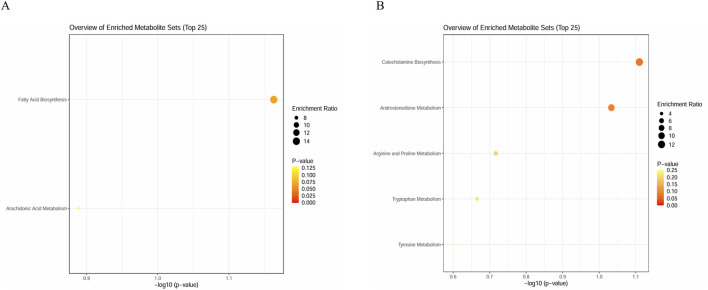
Enrichment analysis plots. **(A)** Enrichment analysis plot of 16 metabolites in serum sample and **(B)** enrichment analysis plot of 11 metabolites in urine samples.

To identify more metabolic pathways, we selected 16 metabolites in serum samples and 11 metabolites in urine samples for enrichment analysis. We queried HMDB for these metabolites and put them into the enrichment module of MetaboAnalyst 6.0 (Table 6). We queried that metabolites in serum samples enrich fatty acid biosynthesis (p = 0.069) and arachidonic acid metabolism (p = 0.129); meanwhile, the metabolites found in urine samples enrich catecholamine biosynthesis (p = 0.078), androstenedione metabolism (p = 0.093), arginine and proline metabolism (p = 0.192), tryptophan metabolism (p = 0.216), and tyrosine metabolism (p = 0.252) (Figure 9). Not all pathways reach statistical significance, but this provides a novel direction for further research to validate. As many similar studies follow a two-phase approach of an initial discovery phase followed by a validation phase, this exploratory metabolomics study still holds significant value.

**TABLE 6:** The HMDB number of 16 serum metabolites and 11 urine metabolites.

Mass-to-charge ratio (*m/z*)	Metabolite	HMDB number
Serum		
246.2441	Myristic acid	HMDB0000806
285.1718	PGP(18:0/18:3(6Z,9Z,12Z))	HMDB0013508
301.2031	TG(14:1(9Z)/16:1(9Z)/20:3n6)	HMDB0047919
318.3017	Decanoylcholine	HMDB0013228
334.2961	MG(i-15:0/0:0/0:0)	HMDB0072878
362.3278	TG(14:0/i-16:0/i-12:0)	HMDB0099868
758.5726	DG(22:5n6/0:0/22:5n6)	HMDB0056359
784.587	PS(18:0/20:0)	HMDB0010164
806.5728	PE(16:0/20:2(11Z:14Z)	HMDB0011510
281.2498	FAHFA(18:1(9Z)/6-O-18:0)	HMDB0112173
339.2343	8,9-Epoxyeicosatrienoic acid	HMDB0002232
369.1748	5a-Dihydrotestosterone sulfate	HMDB0006278
540.3326	LysoPC(0:0/16:0)	HMDB0240262
564.3316	LysoPC(0:0/18:2(9Z,12Z))	HMDB0061700
568.3637	Platelet-activating factor	HMDB0062195
802.5634	PS(22:1(13Z)/15:0)	HMDB0112738
Urine		
221.1656	Tryptamine	HMDB0000303
273.2219	Etiocholanolone	HMDB0000546
285.1716	Deoxycholic acid 3-glucuronide	HMDB0002596
334.296	Margaroylglycine	HMDB0013246
152.0709	Norepinephrine	HMDB0000216
484.2907	Etiocholanolone glucuronide	HMDB0004484
150.0567	4-Hydroxyproline	HMDB0000725
350.0884	Dopamine glucuronide	HMDB0010329
375.1307	Riboflavin	HMDB0000244
449.2552	Pregnanetriol	HMDB0006070
931.5049	5-alpha-Dihydrotestosterone glucuronide	HMDB0006203

Human Metabolome Database, HMDB

### 3.5 Comparison of diagnostic performance of inflammatory markers

**FIGURE 10:**
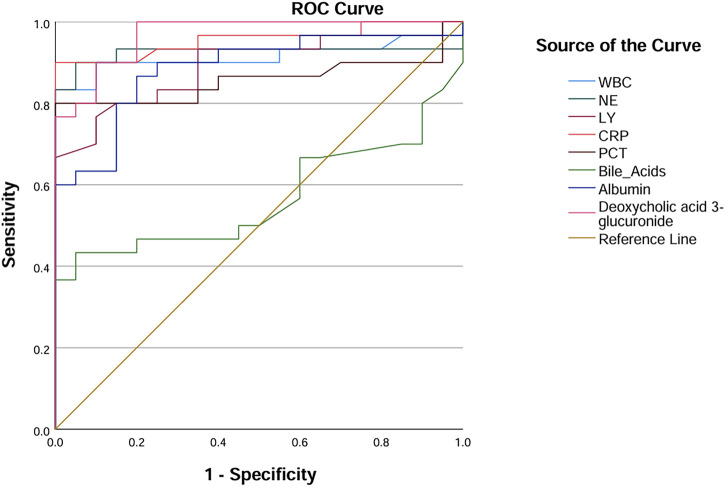
ROC curves of deoxycholic acid 3-glucuronide and traditional inflammation indexes. Abbreviation: WBC, white blood cells; NE, neutrophilicgranulocytes; LY, lymphocytes; CRP, C-reactive protein; PCT, procalcitonin; AUC, area under the curve.

Among the seven metabolic biomarkers, deoxycholic acid 3-glucuronide demonstrated the highest AUC value of 0.968, highlighting its superior diagnostic potential. ROC analysis was conducted to evaluate its diagnostic performance, incorporating traditional inflammatory markers such as PCT, NE, and CRP, as well as statistically significant baseline parameters, including WBC, lymphocyte, bile acids, and albumin. Based on the AUC values, we concluded that deoxycholic acid 3-glucuronide exhibits greater accuracy in identifying IAI than conventional markers, underscoring its significant clinical value (Table 7; Figure 10).

**TABLE 7:** The AUC value of deoxycholic acid 3-glucuronide and traditional inflammatory indexes.

Metabolites	AUC	SE	95% confidence intervals	
			lower	upper
WBC	0.914	0.046	0.825	1.000
NE	0.925	0.046	0.835	1.000
LY	0.891	0.046	0.801	0.981
CRP	0.956	0.029	0.899	1.000
PCT	0.858	0.058	0.744	0.971
Bile Acids	0.568	0.082	0.407	0.728
Albumin	0.885	0.048	0.791	0.979
Deoxycholic acid 3-glucuronide	0.968	0.020	0.929	1.000

Abbreviations: Area under the curve (AUC), C-reactive protein (CRP), Lymphocytes (LY), Neutrophilicgranulocytes (NE), Procalcitonin (PCT), White blood cells (WBC)

### 3.6 Subgroup analysis

**FIGURE 11:**
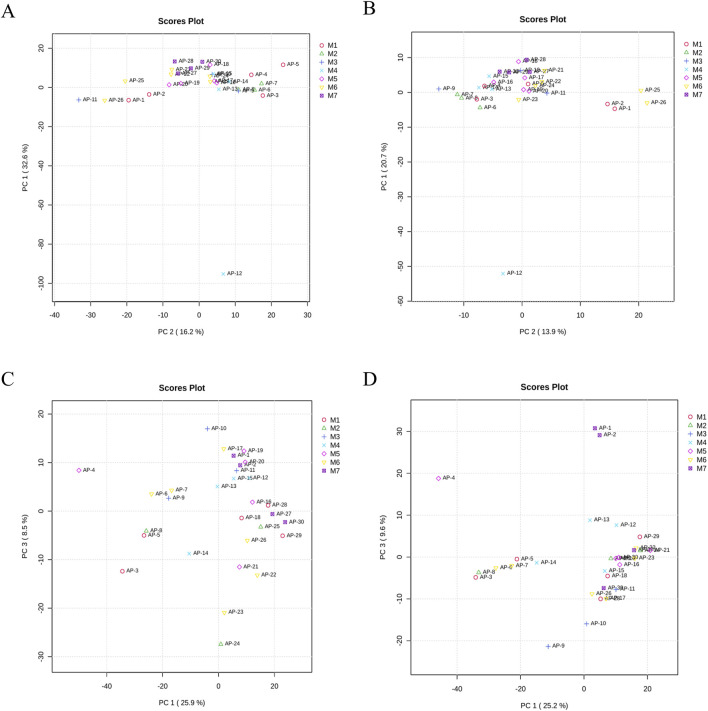
PCA of samples from M1, M2, M3, M4, M5, M6, and M7. **(A)** PCA of serum samples in the positive ion mode, **(B)** PCA of serum samples in the negative ion mode, **(C)** PCA of urine samples in the positive ion mode, and **(D)** PCA of urine samples in the negative ion mode. Each dot represents a single sample. Abbreviation: PCA, principal component analysis; M1, gastric ulcers; M2, duodenal ulcers; M3, small intestinal trauma; M4, colon diverticular perforations; M5, acute non-suppurative appendicitis; M6, acute suppurative appendicitis; and M7 gangrenous perforated appendicitis.

Based on postoperative pathology, the 30 IAI cases were categorized into seven distinct groups, namely five gastric ulcers cases, three duodenal ulcers cases, three small intestinal trauma cases, four colon diverticular perforations cases, five acute non-suppurative appendicitis cases, six acute suppurative appendicitis cases, and four gangrenous perforated appendicitis cases. We performed PCA for these seven groups. However, these groups were not distinctly separated in the PCA plot (Figure 11). This result may be attributed to two main reasons. First, it suggests that IAI caused by different diseases may induce similar metabolic disturbance patterns. Second, based on power analysis, a minimum of 64 samples per group is required to detect subgroup differences with a moderate effect size (d = 0.5), which exceeds the sample size of our current study. Therefore, as an initial investigation, this subgroup analysis cannot rule out the possibility of distinct metabolic features across the seven disease categories.

In this study, the 30 IAI patients were stratified into three groups based on infection severity: Mild infection: seven cases (AP-1, AP-4, AP-6, AP-7, AP-13, AP-15, AP-28); Moderate infection: 18 cases (AP-2, AP-5, AP-8, AP-9, AP-10, AP-11, AP-12, AP-14, AP-16, AP-17, AP-18, AP-19, AP-20, AP-21, AP-22, AP-23, AP-24, AP-25); Severe infection: five cases (AP-3, AP-26, AP-27, AP-29, and AP-30). PCA was performed to assess metabolic differences among the three groups; however, no clear separation was observed, suggesting minimal metabolic variability across the severity groups (Supplementary Figure S2). The potential reasons for this observation are similar to those mentioned earlier. Therefore, this finding does not exclude the possibility of metabolic differences among the severity groups but highlights the need for further investigation with a larger cohort.

## 4 Discussion

To our knowledge, this study may represent the first metabolomic investigation to identify potential serum and urine biomarkers in patients with IAI. Through comprehensive statistical analyses, including PCA, PLS-DA, SVM, and ROC analysis, we identified five serum biomarkers and two urine biomarkers for IAI. The serum biomarkers comprised PGP(18:0/18:3 (6Z,9Z,12Z)), 5α-dihydrotestosterone sulfate, lysoPC(0:0/16:0), lysoPC(0:0/18:2 (9Z,12Z)), and platelet-activating factor. The urine biomarkers included deoxycholic acid 3-glucuronide and margaroylglycine. Compared with the other six metabolites and conventional inflammatory markers, deoxycholic acid 3-glucuronide demonstrated superior diagnostic performance for IAI. Enrichment analysis revealed that 16 serum metabolites were significantly associated with fatty acid biosynthesis and arachidonic acid metabolism, while 11 urine metabolites were predominantly involved in catecholamine biosynthesis and androstenedione metabolism pathways. Notably, our analysis did not reveal distinct metabolic signatures associated with different IAI subtypes or etiological classifications.

Subsequently, we will examine the association between the identified metabolites and inflammatory processes. Preliminary evidence suggests a possible correlation between PGP(18:0/18:3 (6Z,9Z,12Z)) and inflammatory reactivity. PGP(18:0/18:3 (6Z,9Z,12Z)) is a molecular species consisting of a phosphatidylglycerophosphate (PGP) backbone esterified with two fatty acyl chains: a saturated 18:0 (stearic acid) chain and an unsaturated 18:3 (6Z,9Z,12Z) (γ-linolenic acid) chain. As a biosynthetic intermediate, PGP is enzymatically converted to phosphatidylglycerol, a membrane phospholipid that is primarily found in mitochondrial and bacterial membranes (Tague et al., 2019). Furthermore, PGP serves as a crucial biosynthetic precursor for cardiolipin, an essential phospholipid that constitutes the fundamental structural component of the mitochondrial inner membrane (Hryc et al., 2023). Cardiolipin plays a pivotal role in maintaining mitochondrial membrane integrity and facilitating energy metabolism (Schlame and Greenberg, 2017). Importantly, mitochondrial dysfunction, often associated with cardiolipin abnormalities, has been strongly correlated with the pathogenesis of chronic inflammation and oxidative stress (Reynolds et al., 2023). PGP can undergo enzymatic hydrolysis mediated by phospholipase, generating lysophosphatidylglycerophosphate. This bioactive lipid metabolite has been shown to activate immune cells, particularly macrophages, thereby potentially promoting inflammatory responses (Yan and Horng, 2020). Next, stearic acid (18:0), a saturated fatty acid, contributes to the structural integrity and stability of cellular membranes (Demirbolat et al., 2021). In contrast, α-linolenic acid (18:3 (6Z,9Z,12Z)), a polyunsaturated fatty acid with three cis-double bonds, exhibits potential anti-inflammatory properties (Mizuno and Taguchi, 2020). Moreover, PGP(18:0/18:3 (6Z,9Z,12Z)), an omega-3 polyunsaturated fatty acid, serves as a metabolic precursor for the biosynthesis of specialized pro-resolving lipid mediators (SPMs), including resolvins and protectins, which actively participate in the resolution of inflammation through their potent anti-inflammatory and pro-resolving activities (Devassy et al., 2016). PGP(18:0/18:3 (6Z,9Z,12Z)) has been shown to modulate key inflammatory signaling pathways, including NF-κB and MAPK cascades, thereby suppressing the production and release of proinflammatory cytokines such as TNF-α, IL-1β, and IL-6 (Wang et al., 2025). Consequently, PGP(18:0/18:3 (6Z,9Z,12Z)) may exert a dual regulatory effect on inflammatory processes, potentially balancing both proinflammatory and anti-inflammatory responses.

5a-dihydrotestosterone sulfate is a sulfate derivative of 5a-dihydrotestosterone produced through phase II metabolism (Li et al., 2024). The combination of 5α-dihydrotestosterone (5α-DHT) and the androgen receptor can modulate gene expression, thereby influencing the production of inflammatory factors and the function of immune cells (Schiffer et al., 2024). Specifically, 5α-DHT has been shown to inhibit proinflammatory factors such as TNF-α and IL-6, while simultaneously elevating the levels of anti-inflammatory factors like IL-10 (Vojnović Milutinović et al., 2021). 5α-DHT may suppress the NF-κB signaling pathway, leading to a reduction in inflammatory activity (Jain et al., 2012). 5α-DHT can also indirectly regulate inflammation by modulating oxidative stress levels (Mićić et al., 2023). However, the effects of 5α-DHT exhibit tissue specificity. For instance, in the prostate, 5α-DHT promotes the release of inflammatory factors and enhances cell proliferation, contributing to the development of conditions such as benign prostatic hyperplasia (Sánchez-Fernández et al., 2024). Given these complex and context-dependent roles, further research is needed to fully elucidate the involvement of 5α-DHT in inflammation and its associated diseases.

LysoPC(0:0/16:0) and lysoPC(0:0/18:2 (9Z,12Z)) are two important lysophosphatidylcholines (lysoPCs) that play significant roles in cellular processes. The functions of lysoPCs are complex and often exhibit a dual nature. As biologically active lipid molecules, lysoPCs regulate cell signaling and inflammation by interacting with G protein-coupled receptors located on the cell surface (Liu et al., 2020). On the one hand, lysoPCs can promote inflammation by activating the NF-κB and MAPK pathways; on the other hand, they can suppress inflammation by modulating the activity of immune cells (Quan et al., 2016). Furthermore, lysoPC(0:0/16:0) and lysoPC(0:0/18:2 (9Z,12Z)) may exhibit distinct biological functions due to differences in the length and saturation of their fatty acid chains (Foo et al., 2015).

Platelet-activating factor (PAF) is a ubiquitous and potent phospholipid mediator that plays a critical role in the activation and regulation of inflammation, contributing significantly to the pathogenesis of various inflammatory disorders (Liu et al., 2024). PAF activates inflammatory cells such as neutrophils, monocytes, and macrophages, enhancing their functions, including chemotaxis, adhesion, and degranulation (Choi and Ye, 2024). Furthermore, PAF increases vascular permeability, promotes plasma exudation, and amplifies the inflammatory response by inducing the release of proinflammatory factors such as TNF-α, IL-1β, and IL-6 (Starossom et al., 2015). Additionally, PAF is involved in microvascular injury associated with inflammation by promoting platelet activation. It also upregulates the expression of inflammatory genes through the activation of NF-κB and MAPK signaling pathways (Chen et al., 2017). PAF plays a particularly important role in conditions like sepsis, where it synergistically amplifies the inflammatory response by interacting with other inflammatory mediators such as prostaglandins and leukotrienes (Chen et al., 2017). While PAF is predominantly recognized for its strong proinflammatory effects, it may also exhibit certain anti-inflammatory properties when hydrolyzed by PAF acetylhydrolase, which degrades PAF into an inactive form (Chaithra et al., 2018). This dual nature highlights the complex role of PAF in inflammation and underscores its potential as a therapeutic target in inflammatory diseases.

Deoxycholic acid 3-glucuronide (DCA-3G) is a natural human metabolite of deoxycholic acid (DCA), synthesized in the liver through the action of UDP-glucuronosyltransferase. DCA and its metabolites, including DCA-3G, have been implicated in the induction of intestinal inflammation by activating the NF-κB signaling pathway, leading to the upregulation of proinflammatory cytokines such as IL-6 and TNF-α (Mühlbauer et al., 2004). Furthermore, elevated levels of DCA can compromise the integrity of the intestinal epithelial barrier, facilitating bacterial translocation and exacerbating inflammatory responses. Additionally, DCA metabolites may contribute to oxidative stress by promoting the generation of reactive oxygen species (ROS), thereby triggering inflammatory cascades (Huo et al., 2011). Beyond localized effects, DCA metabolites may also play a role in systemic inflammation by modulating lipid metabolism and influencing immune cell function (Cui et al., 2023). Despite these insights, the precise mechanisms underlying the proinflammatory effects of DCA and its metabolites remain incompletely understood, necessitating further research to elucidate their roles in inflammation-related diseases.

Margaroylglycine, an acylglycine characterized by a C-17 fatty acid acyl moiety, is primarily metabolized through amidase-mediated hydrolysis in renal and intestinal tissues, followed by urinary excretion. This metabolite plays a significant regulatory role in inflammatory responses through multiple mechanisms. At the molecular level, margaroylglycine exerts anti-inflammatory effects by inhibiting the NF-κB signaling pathway, downregulating COX-2 expression, and modulating PPARγ activity (Arul Prakash and Kamlekar, 2021). Experimental evidence demonstrates its capacity to reduce proinflammatory mediators, achieving approximately 50% reductions in TNF-α, IL-6, and PGE2 levels (Leung et al., 2020). The compound’s immunomodulatory properties extend to cellular regulation, where it effectively suppresses M1 macrophage polarization, maintains T lymphocyte subset homeostasis, and attenuates neutrophil activity (Gan et al., 2021). Furthermore, margaroylglycine exhibits tissue-protective effects by preserving endothelial barrier integrity, inhibiting fibroblast activation, and mitigating epithelial inflammatory responses (Satyanarayana et al., 2021). These multifaceted actions position margaroylglycine as a potential endogenous anti-inflammatory factor within the inflammatory microenvironment, offering therapeutic potential for inflammation-related pathologies.

Emerging evidence suggests significant crosstalk between inflammatory responses and multiple metabolic pathways, particularly fatty acid biosynthesis, arachidonic acid metabolism, catecholamine biosynthesis, and androstenedione metabolism, which collectively contribute to regulating immune homeostasis and inflammatory processes. Inflammation induces systemic metabolic reprogramming in organisms, significantly elevating the importance of fatty acid biosynthesis. This metabolic pathway demonstrates a dual role in inflammatory regulation: (1) it promotes proinflammatory responses through saturated fatty acid-mediated activation of the TLR4/NF-κB signaling pathway, NLRP3 inflammasome activation, and upregulation of proinflammatory cytokines, including TNF-α and IL-6 (Rocha et al., 2016); (2) conversely, it exerts anti-inflammatory effects through the production of specialized pro-resolving mediators, activation of the PPARγ signaling pathway, and suppression of NF-κB signal transduction (Angela et al., 2016).

Arachidonic acid metabolism constitutes a sophisticated biochemical network comprising three major enzymatic pathways: (1) the cyclooxygenase pathway, responsible for the production of prostaglandin (PG) and thromboxane (TX) (Kaur et al., 2019); (2) the lipoxygenase pathway, generating leukotrienes (LTs), hydroxyeicosatetraenoic acids (HETEs), and lipoxins (LXs) (Pang et al., 2022); and (3) the cytochrome P450 pathway, producing epoxyeicosatrienoic acids (EETs) and hydroxyeicosatetraenoic acids (HETEs) (Trostchansky et al., 2019). These metabolic derivatives play pivotal roles in inflammatory regulation, where PGE2 mediates vasodilation and pain sensation, LTB4 acts as a potent chemotactic factor, and TXA2 promotes platelet aggregation, while LXs facilitate inflammation resolution, resolvins modulate immune responses, and protectins exert cytoprotective effects (Hong et al., 2023). Beyond these inflammatory mediators, arachidonic acid metabolites also significantly influence multiple signaling pathways, including proinflammatory cascades such as NF-κB, MAPK, and PI3K/Akt, as well as anti-inflammatory pathways including PPARγ and Nrf2 (Wang B. et al., 2021).

Catecholamine biosynthesis demonstrates significant involvement in acute inflammatory conditions, particularly sepsis, through its production of key hormones, including dopamine, norepinephrine, and epinephrine. These hormones exert their biological effects *via* specific receptor binding, leading to activation of the sympathetic nervous system, initiation of vagus nerve reflexes, and modulation of neurotransmitter release. Furthermore, catecholamines significantly influence immune cell functions, regulating macrophage polarization patterns, T lymphocyte differentiation processes, and dendritic cell maturation states. The complex interplay between these hormonal signals and immune cell activities collectively modulates inflammatory responses and determines disease progression. Furthermore, androstenedione metabolism serves as a crucial precursor for testosterone and estrone biosynthesis. Testosterone exerts anti-inflammatory effects through multiple mechanisms, including suppression of IL-1β and TNF-α production, enhancement of IL-10 secretion, and modulation of TGF-β expression (Filippi et al., 2019). Concurrently, estrone demonstrates potent immunomodulatory properties by inhibiting NF-κB nuclear translocation, upregulating IκBα expression, and regulating miRNA profiles (Kumar and Goyal, 2021). These steroid hormone-mediated regulatory effects collectively contribute to the mitigation of cytokine storms, preservation of intestinal barrier integrity, improvement of organ function, and, ultimately, reduction of mortality rates in acute inflammatory conditions.

We observed a predominant downward trend in metabolite levels, except deoxycholic acid 3-glucuronide, which may be attributed to two primary mechanisms: (1) substantial consumption of metabolic substrates for inflammatory factor production and (2) negative feedback regulation within the inflammatory signaling network (Takeda et al., 2023).

Finally, several limitations should be acknowledged in our current study. First, our cohort does not encompass the full spectrum of IAI etiologies, notably excluding conditions such as acute cholecystitis and acute pancreatitis. Second, a prospective case–control to assess the biomarkers' performance in a real-world clinical setting is absent. Third, it is important to note that our SVM-DA may be subject to overfitting, as the same samples were used for both metabolite selection and classification. Due to the limited sample size, we were unable to perform independent validation or cross-validation. Therefore, the results should be interpreted as preliminary and require confirmation in future studies with larger cohorts and more robust analytical approaches. Fourth, the current study design, with samples collected 1 hour before surgery, limits the generalizability of our findings to a broader clinical context. Future prospective case–control studies in patients with suspected IAI at ICU admission, compared to matched controls, are needed to determine the broader clinical utility of these metabolites. Fifth, our study only focuses on the metabolic differences between healthy people and people with IAI but overlooks differentiating IAI biomarkers attributed to specific etiology by comparing IAI patients with those who present the same underlying conditions (for example, appendicitis or perforation) but do not develop the IAI, which may increase the metabolic “noise” derived from the underlying pathology and limit the clinical application of our outcomes. In the future, we will include more participants and initiate a multicenter project involving hospitals of different grades to dynamically monitor changes in metabolites at various stages of a specific disease, which may validate our current findings and identify more biomarkers with clinical value.

## 5 Conclusion

This study identified five serum and two urine metabolites as potential biomarkers for early detection of IAI in emergency settings. These findings represent a significant advancement in rapid IAI diagnosis, which is crucial for timely treatment initiation and improved outcomes. Further validation in larger, diverse cohorts is needed to confirm their clinical utility.

## Data Availability

The original contributions presented in the study are publicly available. This data can be found here: https://figshare.com/s/b70fc11ca57e45dc4429.

## Supplementary material

SUPPLEMENTARY FIGURE S1
TIC chromatograms of metabolites of urine samples from the healthy group and the IAI group. **(A)** TIC profiles of the healthy group in the positive ion mode; **(B)** TIC profiles of the IAI group in the positive ion mode; **(C)** TIC profiles of the healthy group in the negative ion mode; **(D)** TIC profiles of the IAI group in the negative ion mode. Red arrows indicate metabolites subsequently identified as having different abundances in the two groups. Abbreviations: TIC, total ion chromatography; IAI, acute intra-abdominal infection.

SUPPLEMENTARY FIGURE S2
PCA of samples from B1, B2, and B3. **(A)** PCA of serum samples in the positive ion mode; **(B)** PCA of serum samples in the negative ion mode; **(C)** PCA of urine samples in the positive ion mode; **(D)** PCA of urine samples in the negative ion mode. Each dot represents a single sample. Abbreviation: PCA, principal component analysis. IAI, Acute intra-abdominal infection. B1, mild IAI; B2, moderate IAI; B3, severe IAI.

SUPPLEMENTARY FIGURE S3
The informed consent used in this study.
