# Supplementary figures and images for: Untargeted metabolomics for acute intra-abdominal infection diagnosis in serum and urine using UHPLC-TripleTOF MS
Source: Front Mol Biosci. 2025 May 8;12:1534102. doi: 10.3389/fmolb.2025.1534102 (PMC12094940; doi:10.3389/fmolb.2025.1534102)

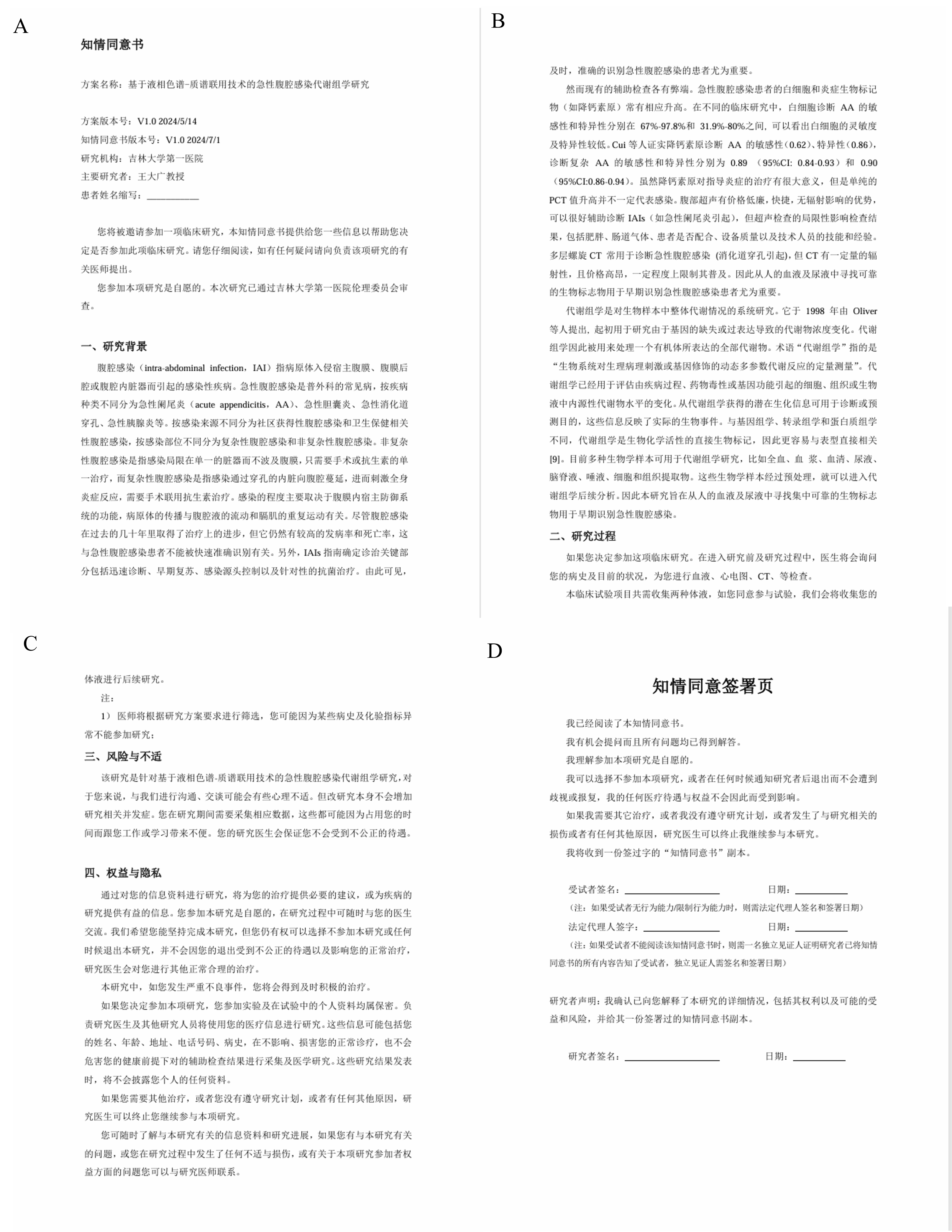

Supplement: Supplementary file 1 [file Image3.tif]

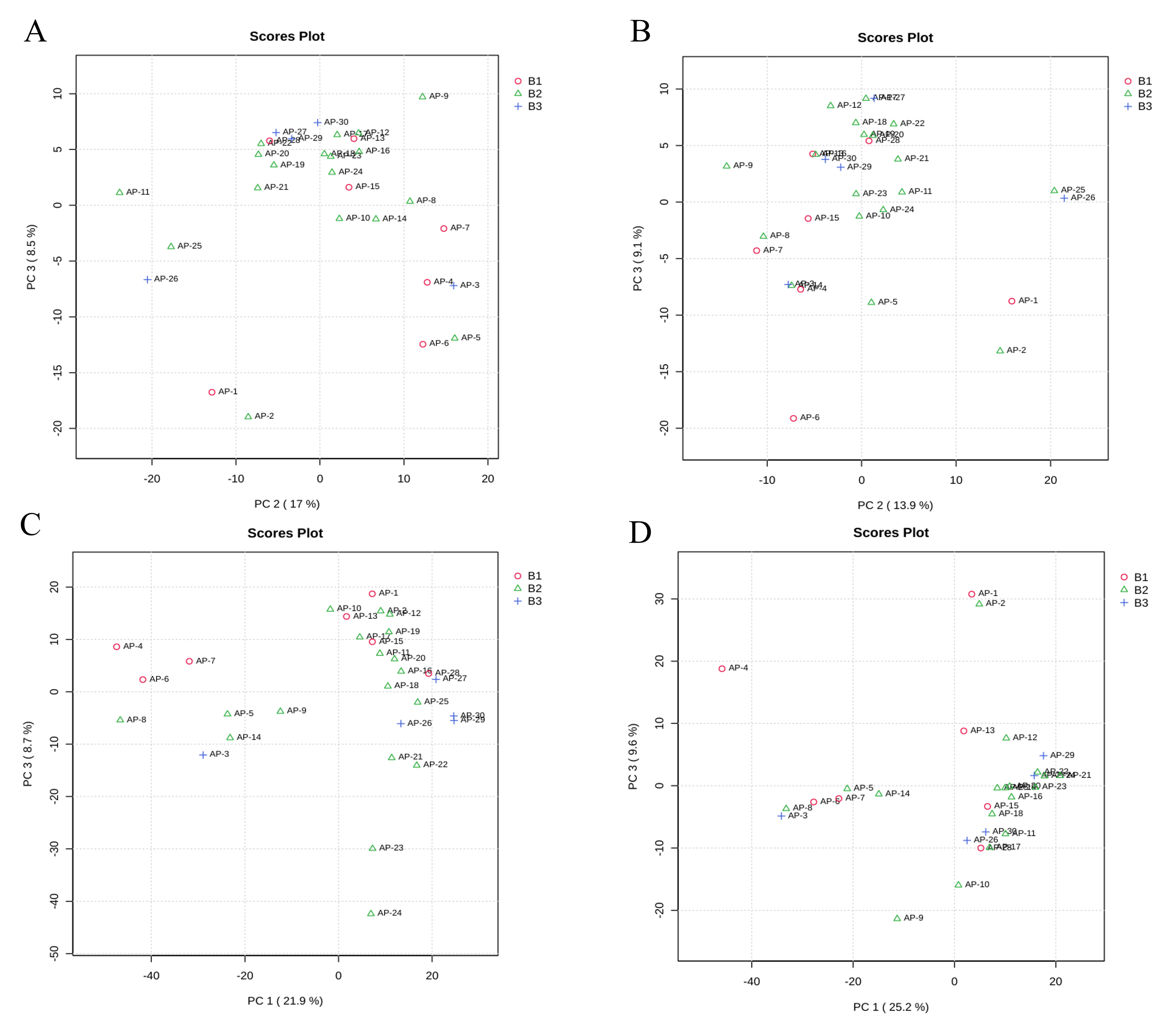

Supplement: Supplementary file 3 [file Image2.tif]

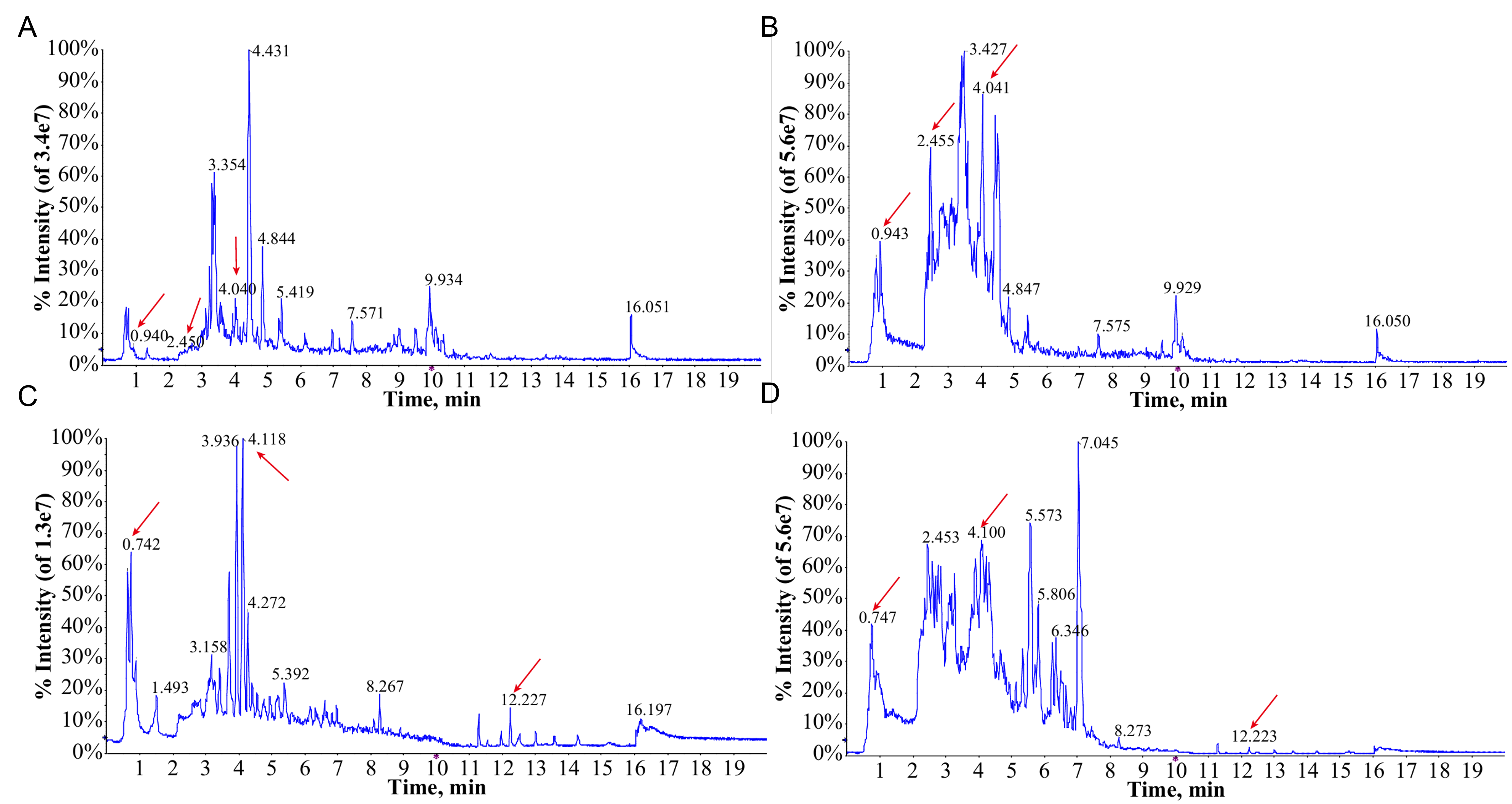

Supplement: Supplementary file 4 [file Image1.tif]
